# Short-term versus long-term psychotherapy for adult psychiatric disorders: a protocol for a systematic review with meta-analysis and trial sequential analysis

**DOI:** 10.1186/s13643-019-1099-0

**Published:** 2019-07-13

**Authors:** Sophie Juul, Stig Poulsen, Susanne Lunn, Per Sørensen, Janus Christian Jakobsen, Sebastian Simonsen

**Affiliations:** 1 Stolpegaard Psychotherapy Centre, Mental Health Services, Capital Region of Denmark, Stolpegaardsvej 20, 2820 Gentofte, Denmark; 2 Department of Psychology, University of Copenhagen, Østre Farimagsgade 2A København K, 1353 Copenhagen, Denmark; 3 Copenhagen Trial Unit, Tagensvej 22, København N, 2200 Copenhagen, Denmark; 4 Department of Cardiology, Holbæk Hospital, Smedelundsgade 60, 4300 Holbæk, Denmark

**Keywords:** Psychotherapy duration, Psychiatric disorders, Short-term psychotherapy, Long-term psychotherapy, Dose-effect

## Abstract

**Background:**

Psychiatric disorders are highly prevalent and associated with great symptomatic, functional, and health economic burdens. Psychotherapy is among the recommended and used interventions for most psychiatric disorders and is becoming widely accessible in mental health systems. The effects of specific forms of psychotherapy (e.g., psychodynamic therapies, cognitive and behavioral therapies, humanistic therapies, and systemic therapies) have been assessed previously in systematic reviews, but the appropriate psychotherapy duration for psychiatric disorders has not been reviewed. The aim of this systematic review will be to synthesize the evidence of the effects of short-term compared with long-term psychotherapy for all adult psychiatric disorders.

**Methods/design:**

A comprehensive search for relevant published literature will be undertaken in Cochrane Central Register of Controlled Trials (CENTRAL), Medical Literature Analysis and Retrieval System Online (MEDLINE), Excerpta Medica database (EMBASE), Latin American and Caribbean Health Sciences Literature (LILACS), PsycINFO, Science Citation Index Expanded (SCI-EXPANDED), Social Sciences Citation Index (SSCI), Conference Proceedings Citation Index—Science (CPCI-S), and Conference Proceedings Citation Index—Social Science & Humanities (CPCI-SSH) to identify relevant trials. We will search all databases from their inception to the present. We will include randomized clinical trials comparing a short-term and a long-term version of the same psychotherapy type for adult psychiatric disorders including attention deficit hyperactivity disorder, psychotic disorders, depressive disorders, bipolar disorders, anxiety disorders, obsessive-compulsive disorder, trauma- and stressor-related disorders, eating disorders, and personality disorders (as defined by standardized diagnostic criteria). We will rely on the trialists defining their compared interventions as short term and long term (or similar terminology). Primary outcomes will be quality of life, serious adverse events, and symptom severity. Secondary outcomes will be suicide or suicide attempts, self-harm, and level of functioning. Two review authors will independently extract data and perform risk of bias assessment using the Cochrane risk of bias tool. A meta-analysis will be performed as recommended by the Cochrane Handbook for Systematic Review of Interventions, bias will be assessed with domains, and Trial Sequential Analysis will be conducted to control random errors. Certainty of the evidence will be assessed by GRADE.

**Discussion:**

As psychotherapy is among the treatments of choice for most adult psychiatric disorders, a systematic review evaluating the benefits and harms of short-term compared with long-term psychotherapy is urgently needed. It is the hope that this review will be able to inform best practice in treatment and clinical research of these highly prevalent and burdensome disorders.

**Systematic review registration:**

PROSPERO CRD42019128535

**Electronic supplementary material:**

The online version of this article (10.1186/s13643-019-1099-0) contains supplementary material, which is available to authorized users.

## Background

It is estimated that each year, 38.2% of the European population suffer from a psychiatric disorder [1]. The economic burden from psychiatric disorders is excessive, not only because of high direct health care costs, but also because of indirect costs like sick days, disability, and early retirement [1]. Psychotherapy is among the recommended and widely used interventions for most disorders [2]. Specific types of psychotherapy have already been systematically reviewed, but the appropriate length of psychotherapy for all adult psychiatric disorders has not been reviewed previously. To present a complete overview of the evidence and to increase the statistical power, we will therefore in the present review include any adult psychiatric disorder. The major categories of adult psychiatric disorders listed in the *Diagnostic and Statistical Manual of Mental Disorders*, *5th edition* (DSM-V) [3] are the following.

*Attention Deficit Hyperactivity Disorder* (*ADHD*) is characterized by a persistent pattern of inattention and/or hyperactivity and impulsivity that significantly interferes with functioning and development [3]. ADHD is one of the most common psychiatric disorders of childhood and adolescence, and it often persists into adulthood. The predominant characteristics of adult ADHD differ from typical ADHD characteristics in children. Symptoms of hyperactivity or impulsivity are typically less obvious in adults, whereas symptoms of inattention are more dominant [4]. Epidemiologic studies of adult ADHD have estimated the current prevalence to be 4.4% in the USA and 3.4% internationally [4, 5]. The total economic burden of ADHD in America has been estimated to be 31.6 billion US dollars in 2010 [6] including both direct costs, other health care costs, health care costs for family members, and work loss of patients and their relatives.

*Psychotic disorders* are characterized by abnormalities in one or more of the following five domains: delusions, hallucinations, disorganized thinking, disorganized or abnormal motor behavior, and negative symptoms [3]. The estimated annual prevalence of all psychotic disorders is 2.6% [7]. The most common psychotic disorder is schizophrenia with an estimated median lifetime prevalence of 4.0 per 1000 and a lifetime morbid risk of 7.2 per 1000 [8]. Annual costs for the schizophrenia population have been systematically reviewed and estimated to range from 94 million to 102 billion US dollars. Indirect costs contributed to 50–85% of the total costs associated with schizophrenia [9].

*Bipolar disorders* are characterized by serious mood changes involving mood elevation (mania or hypomania) either alone or followed by major depressive episodes [3]. Bipolar disorder subtypes include bipolar I and bipolar II. Bipolar I disorder is associated with manic episodes nearly always followed by major depressive and hypomanic episodes. Bipolar II disorder is associated with at least one hypomanic episode, at least one major depressive episode, and the absence of manic episodes. The international annual prevalence is estimated to be 0.4% for bipolar I disorder and 0.3% for bipolar II disorder [10]. In 2009, the estimated annual direct and indirect costs of bipolar I and II disorders were 30.7 and 120.3 billion US dollars, respectively [11].

*Depressive disorders* are characterized by the presence of a sad, empty, and irritable mood often accompanied by somatic and cognitive changes resulting in significant functional impairment [3]. The most common depressive disorder is major depressive disorder (unipolar depression) with an annual prevalence of approximately 7% both in Europe [1] and in the USA [12]. The estimated annual economic burden of adults with major depressive disorder, including direct medical costs, workplace costs, and costs associated with comorbidities exceeded 200 billion US dollars [13] in the USA in 2010.

*Anxiety disorders* are characterized by excessive and counterproductive feelings of fear and anxiety often accompanied by behavioral disturbances such as pervasive avoidance behaviors [3]. Different anxiety disorders exist, which differ from one another in the types of objects or situations that induce hyperarousal or avoidance behavior [3]. The prevalence of anxiety disorders is estimated to be 18% in the USA [14] and 14% in European countries [1], placing them among the most prevalent psychiatric disorders worldwide. Costs associated with anxiety disorders have previously been reported to be 46.6 billion US dollars in the USA [15] including both direct and indirect costs.

*Obsessive*-*compulsive disorder* (*OCD*) is characterized by recurrent intrusive thoughts, images, or urges (obsessions) with or without repetitive mental or behavioral acts (compulsions) [3]. OCD among adults has an annual prevalence of 1.2% and a lifetime prevalence of 2.3% [16, 17]. The annual economic burden of OCD is estimated to be 2272 euros per patient when including both direct and indirect costs [18].

*Trauma*- *and stressor*-*related disorders* are characterized by psychological distress following exposure to a traumatic or stressful event. The most common trauma disorder is post-traumatic stress disorder (PTSD) [3]. PTSD is a prevalent and disabling disorder associated with delayed help seeking [19]. The estimated annual prevalence of PTSD is 2% in Europe [1] and 4.7% in the USA [20], and the estimated lifetime prevalence is 3.9% across 26 countries ranging from low to high income [21]. The total costs of PTSD per patient have been estimated to 1082 million euros including both direct and indirect costs [18].

*Eating disorders* are characterized by a persistent disturbance in eating behavior resulting in altered consumption or absorption of food that significantly impairs health and psychosocial functioning [3]. The most common eating disorders are anorexia nervosa, bulimia nervosa, and binge-eating disorder. Lifetime prevalence of anorexia nervosa, bulimia nervosa, and binge-eating disorder are estimated to be 0.9, 1.5, and 3.5%, respectively, among women and 0.3, 0.5, and 2.0%, respectively, among men [22]. The estimated annual prevalence of eating disorders is 0.9% in the European population [1]. Annual costs per patient are estimated to range from 1288 to 8042 US dollars [23].

*Personality disorders* are characterized by enduring and inflexible patterns of emotional, behavioral, and interpersonal problems that deviate markedly from cultural expectations. According to DSM-V, the following nine personality disorders exist. Personality disorders onset in adolescence or early adulthood and are associated with great psychosocial distress and impairment [3]. In a systematic review of the economic burden of personality disorders, the estimated direct and indirect costs were 11,126 euros for patients 12 months prior to seeking treatment. Direct medical costs accounted for two thirds of these costs, while the remaining costs were related to productivity losses [24].

### Description of the interventions

Different schools of psychotherapy exist. They are often divided into the following categories: *psychodynamic therapies*, *cognitive and behavioral therapies*, *humanistic therapies*, and *systemic therapies* [2].

*Psychodynamic* (*or psychoanalytical*) *therapies* encompass the many approaches that are influenced by Freud’s psychoanalysis but have developed into different independent schools [2]. Traditionally, psychodynamic therapies have been considered as long-term therapies, perhaps due to the notion that the uncovering of unconscious emotions and conflicts cannot be achieved with a fixed time limit [25]. Long-term psychoanalytical psychotherapy has previously been systematically reviewed yielding different results [26, 27]. Today, different lengths of psychodynamic therapies have been developed to treat different forms of psychopathology. In addition to traditional psychoanalysis, examples of long-term psychodynamic treatments are transference-focused psychotherapy (TFP), a psychodynamic treatment rooted in object relations theory lasting up to 3 years [28, 29], and mentalization-based therapy [30], an 18-month psychodynamic treatment rooted in attachment theory. Both are developed specifically to treat borderline personality disorder. Further, different variations of short-term psychodynamic therapy have been developed to treat a variation of common psychiatric disorders, most notably anxiety disorders, depressive disorders, certain behavior disorders, and personality disorders [31]. Short-term psychodynamic therapies vary in treatment duration but typically last between 12 and 24 sessions [31].

*Cognitive and behavioral therapies* (CBT) encompass many integrative approaches. Historically, behavior therapy (first wave CBT) developed from the learning theories of Pavlov [32] and Skinner [33]. An integration of a cognitive component to classical behavioral theories was first established by Beck [34, 35], who developed what is now often referred to as second-wave CBT. CBT is now often delivered as a short-term treatment, typically lasting between 12 and 20 sessions, for a variation of common psychiatric disorders like depressive disorders [34], anxiety disorders [36], obsessive-compulsive disorder [37], personality disorders [38], and eating disorders [39]. Different durations of CBT are also available for the treatment of schizophrenia [40, 41]. Today, so-called third-wave cognitive therapies have emerged, characterized by more integrative approaches to psychotherapy, incorporating techniques from Buddhist mindfulness, psychodynamic therapies, or Gestalt therapy [2]. These include dialectical behavior therapy [42] and schema-focused therapy (SFT) [29, 43], which are both long-term therapies for borderline personality disorder (up to 3 years), and acceptance and commitment therapy (ACT) [44] and compassion-focused therapy (CFT) [45], which are often delivered as short-term treatments for various psychiatric disorders [46, 47].

*Humanistic therapies* are characterized by psychotherapy approaches derived from humanistic and existentialist philosophy. Major approaches within this orientation are person-centered therapy [48], Gestalt therapy [49], existential psychotherapy [50], and process-experiential/emotion-focused therapy [51]. All humanistic therapies share the notion of empathic understanding, the promotion of in-therapy experiencing, and a belief in the uniquely human growth tendency by applying a consistent person-centered view involving concern for each patient’s individual experience and differing needs [2]. Humanistic therapies have not been developed to treat specific types of disorders and are traditionally considered open-ended, which is also aligned with the person-centered way of thinking. However, different lengths of humanistic therapies have been studied, e.g., PE-EFT as a short-term treatment (down to 5 weeks) for depressive disorders [52] and as a 20-week treatment for trauma-related disorders [53].

*Systemic therapies* are characterized by a systemic approach to psychotherapy defining patients’ problems as contextually rather than individually derived. Most often, the context of interest is the partner or the family, but it can also be a broader context, such as the extended family or a classroom [2]. Different systemic therapies exist for different types of psychopathology. Examples are family-based therapy for eating disorders [54], attachment-based family therapy for depressed adolescents (ABFT) [55], parent management training for childhood conduct disorders [56], psychoeducational family interventions for schizophrenia [57] and bipolar disorder [58], and systemic treatments for substance-use disorders [59, 60]. Different lengths of systemic therapies exist. However, the typical duration is between 10 and 25 sessions.

Other forms of psychotherapy exist, e.g., interpersonal therapy (IPT) [61] or cognitive-analytic therapy (CAT) [62]. However, it is beyond the scope of this review to mention all new approaches to psychotherapy since the field is constantly expanding. Further, despite the existence of well-established manualized and evidence-based approaches to psychotherapy, a large proportion of practicing psychotherapists define themselves as eclective or integrative [63].

### How the interventions might work

It is a common opinion among clinicians and researchers that patients suffering from complex psychiatric distress require longer and more intensive psychotherapy [27]. Complex psychiatric distress can be defined as disorders, which by definition are enduring and inflexible [27], such as personality disorders or schizophrenia, chronic psychiatric disorders (defined as lasting at least a year), or multiple psychiatric disorders. A related assumption is that complex and severe problems typically take longer to improve than less complex or acute psychiatric distress [25, 64]. This is due to the inherent inflexibility of the psychopathology and the complexity of the required therapeutic techniques. Such potential therapeutic techniques could be provocation of affect or working with the therapeutic alliance [25]. These are techniques that are potentially hard to carry out when faced with time constraints. However, it is often argued that such techniques are essential to effective psychotherapy [65].

In contrast, one could argue that long-term therapies can become counterproductive, given that the same therapeutic techniques will be repeated for a long period of time without continuous assessment of their effects. It is possible that given the limited therapeutic time, planned short-term psychotherapy forces both patients and therapists to establish and maintain a focus throughout the treatment process [66]. Further, issues regarding termination of treatment are particularly important when conducting short-term psychotherapy, where concerns about termination are, almost by definition, always present [67, 68]. Thus, a possible advantage of short-term therapies is that both therapist and patient are forced to address difficult themes associated with separation and loss from the very beginning instead of postponing them for later.

### Why is it important to do this review?

It is essential to investigate the optimal duration of psychotherapy for psychiatric disorders, because of the potential patient and health economic burden from long-term psychotherapy and because of the potential harmful effects of terminating treatment prematurely [69]. If short-term psychotherapy is the optimal treatment approach, then this could result in a reduction of waitlists and thus a greater access to evidence-based care. On the contrary, if long-term psychotherapy is the most optimal treatment, then it becomes sensible for mental health systems to invest in these treatments, as they would translate into greater health and occupational benefits [70].

The relationship between the number of sessions (dose) and patient improvement (effect) in psychotherapy has previously been studied with mixed results [70, 71]. There are studies indicating that increased number of sessions is associated with diminishing results [72]. There are also studies indicating that the speed of improvement is dependent on patients pretreatment functioning [73] and that some patients require different dosages to receive the same effect. However, most research on the association between dose and effect is based on uncontrolled studies [70–72, 74, 75] which can only show that patients improve during treatment. Whether this improvement can be attributed to the treatment, can only be established with randomized controlled trials, in which shorter and longer therapies are directly compared. A systematic review of such randomized clinical trials might allow us to assess the safety profile of the different treatment options directly. We are already aware of two randomized clinical trials comparing a short-term and a long-term version of the same psychotherapy type for one or more adult psychiatric disorders [76, 77]. We have performed a preliminary literature search in the Cochrane Database of Systematic Reviews (search terms, short-term or brief and long-term or standard psychotherapy) for previous systematic reviews comparing a short-term and a long-term version of the same psychotherapy type for one or more adult psychiatric disorders. We identified 1114 hits. From this preliminary literature search, we have only identified one empty systematic review [78].

The present systematic review aims at forming the basis for evidence-based guideline recommendations for the optimal duration of psychotherapy for adult psychiatric disorders taking bias risk (systematic errors), play of chance (random errors), and certainty of the findings into consideration. The objective of this review will be to assess the beneficial and harmful effects of short-term psychotherapy compared with long-term psychotherapy for adult psychiatric disorders.

## Methods

The present protocol has been registered in the PROSPERO database (registration number, CRD42019128535) and is being reported in accordance with the reporting guidance provided in the Preferred Reporting Items for Systematic Reviews and Meta-Analysis Protocols (PRISMA-P) statement [79, 80] (see checklist in Additional file 1).

### Criteria for considering studies for this review

#### Types of studies

We will include randomized clinical trials irrespective of trial design, setting, publication status, publication year, and language. We will not include quasi-randomized trials and observational studies.

#### Types of participants

Adults (as defined by trialists) with a primary diagnosis of any of the following psychiatric disorders: attention deficit hyperactivity disorder, psychotic disorders, depressive disorders, bipolar disorders, anxiety disorders, obsessive-compulsive disorder, trauma- and stressor-related disorders, eating disorders, and personality disorders, as defined by standardized diagnostic criteria from either ICD-10 [81], DSM-5 [3], or earlier versions (ICD-10 codes: F20–29, F30–39, F40–49, F50–59, F60–69, and F90–90.9). Participants will be included irrespective of sex and comorbidities.

#### Types of interventions

Experimental group: we will accept any type of short-term psychotherapy (or similar terms used by the trialists).

Control group: we will accept any type of long-term psychotherapy (or similar terms used by the trialists).

We will rely on the trialists defining their compared interventions as short-term and long-term (or similar terminology). We will include trials comparing a short-term and a long-term version of the same psychotherapy type (e.g., short-term psychodynamic therapy compared to long-term psychodynamic therapy). We will not include trials comparing short-term psychotherapy (e.g., short-term cognitive behavioral therapy) with a different type of psychotherapy (e.g., long-term psychodynamic therapy) delivered as long-term therapy. Further, we will include trials with the same dose (sessions) but with different frequencies, e.g., 12 sessions delivered over 6 weeks compared to 12 sessions delivered over 12 weeks.

### Outcome measures

#### Primary outcomes

1. Quality of life (continuous data)
2. Serious adverse events (dichotomous data). We will use the International Conference on Harmonisation of technical requirements for registration of pharmaceuticals for human use—Good Clinical Practice (ICH-GCP) definition of a serious adverse event, which is any untoward medical occurrence that resulted in death, was life-threatening, required hospitalization or prolonging of existing hospitalization and resulted in persistent or significant disability or jeopardized the patient [82]. If the trialists do not use the ICH-GCP definition, we will include the data if the trialists use the term “serious adverse event.” If the trialists do not use the ICH-GCP definition nor use the term serious adverse event, then we will also include the data, if the event clearly fulfills the ICH-GCP definition for a serious adverse event.
3. Symptom severity assessed by any valid disease-specific symptom scale (continuous data). Symptoms will be analyzed separately for each disorder.

#### Secondary outcomes

1. Suicide or suicide attempts as defined by trialists (dichotomous data)
2. Self-harm as defined by trialists (dichotomous data)
3. Level of functioning as defined by trialists (continuous data)

#### Assessment time points

The primary assessment time point will be the time point closest to the end of treatment in the trials’ long-term intervention group for all outcomes. For example, if a trial compares a 6-month and a 12-month version of the same psychotherapy type and outcomes are assessed every second month throughout the trial, we will select the assessment time point closest to the end of the 12-month intervention as the primary assessment time point for all outcomes. We will secondarily assess all outcomes at maximum follow-up if longer term follow-up is assessed.

### Search methods for identification of studies

#### Electronic searches

We will search Cochrane Central Register of Controlled Trials (CENTRAL), Medical Literature Analysis and Retrieval System Online (MEDLINE), Excerpta Medica database (EMBASE), Latin American and Caribbean Health Sciences Literature (LILACS), PsycINFO, Science Citation Index Expanded (SCI-EXPANDED), Social Sciences Citation Index (SSCI), Conference Proceedings Citation Index—Science (CPCI-S), and Conference Proceedings Citation Index—Social Science & Humanities (CPCI-SSH) to identify relevant trials. We will search all databases from their inception to the present. For a detailed search strategy for all electronic databases, see Additional file 2. The search strategy for PsycINFO will be given at the review stage.

#### Searching other resources

The reference lists of relevant publications will be checked for any unidentified randomized trials. We will contact the authors of included studies by email asking for unpublished randomized trials. Further, we will search for ongoing trials on the following:

- ClinicalTrials.gov (www.clinicaltrials.gov)
- Google Scholar (https://scholar.google.dk/)
- The Turning Research into Practice (TRIP) Database (https://www.tripdatabase.com/)
- European Medicines Agency (EMA) (http://www.ema.europa.eu/ema/)
- US Food and Drug Administration (FDA) (www.fda.gov)
- China Food and Drug Administration (CFDA) (http://eng.sfda.gov.cn/WS03/CL0755/)
- Medicines and Healthcare Products Regulatory Agency (https://www.gov.uk/government/organisations/medicines-and-healthcare-products-regulatoryagency)
- The World Health Organization (WHO) International Clinical Trials Registry Platform (ICTRP) search portal (http://apps.who.int/trialsearch/)
- Cochrane Database of Systematic Reviews
- http://www.evidencebasedpsychotherapies.org/index.php?id=25

Additionally, we will hand search conference abstracts from psychiatry conferences for relevant trials. We will also consider relevant-for-the-review unpublished and gray literature trials if we identify these.

## Data collection and analysis

We will perform the review following recommendations of the Cochrane Collaboration [83]. The analyses will be performed using Trial Sequential Analysis [84] and Stata version 16 (StataCorp LLC, College Station, TX, USA) [85].

### Selection of studies

Two authors (SJ and SS) will independently screen titles and abstracts. We will retrieve all relevant full-text study reports/publications, and two review authors (SJ and SS) will independently screen the full text and identify and record reasons for exclusion of the ineligible studies. We will resolve any disagreement through discussion, or if required, we will consult a third person (JCJ). Trial selection will be displayed in an adapted flow diagram as per the Preferred Reporting Items for Systematic Reviews and Meta-Analyses (PRISMA) statement [86].

### Data extraction and management

Two authors (SJ and SS) will independently extract data from included trials. Disagreements will be resolved by discussion with a third author (JCJ). We will assess duplicate publications and companion papers of a trial together to evaluate all available data simultaneously (maximize data extraction, correct bias assessment). We will contact the trial authors by email to specify any additional data, which may not have been reported sufficiently or at all in the publication.

#### Trial characteristics

We will extract the following data: bias risk components (as defined below), trial design (parallel, factorial, or crossover), number of intervention arms, length of follow-up, estimation of sample size, and inclusion and exclusion criteria.

#### Participant characteristics and diagnosis

We will extract the following data: number of randomized participants, number of analyzed participants, number of participants lost to follow-up/withdrawals/crossover, compliance with interventions, age range (mean or median), sex ratio, and type of psychiatric disorder.

We will additionally report the proportion of participants in the compared groups who receive psychotropic medication.

#### Short-term psychotherapy characteristics

We will extract the following data: short-term psychotherapy type, treatment duration, number of sessions (dose), session lengths (minutes), number of sessions per week, and treatment format.

#### Long-term psychotherapy characteristics

We will extract the following data: long-term psychotherapy type, treatment duration, number of sessions (dose), session lengths (minutes), number of sessions per week, and treatment format.

#### Co-intervention characteristics

We will extract the following data: type of co-intervention, treatment duration of co-intervention, number of sessions (or dose), and treatment format.

#### Outcomes

All outcomes listed above will be extracted from each randomized clinical trial, and we will identify if outcomes are incomplete or selectively reported according to the criteria described later in “incomplete outcome data” bias domain and “selective outcome reporting” bias domain.

#### Notes

Funding of the trial and notable conflicts of interest of trial authors will be extracted, if available. We will note in the “Characteristics of included studies” table if outcome data were not reported in a usable way. Two review authors (SJ and SS) will independently transfer data into the Stata file [85]. Disagreements will be resolved through discussion, or if required, we will consult with a third author (JCJ).

#### Assessment of risk of bias in included studies

We will use the instructions given in the *Cochrane Handbook for Systematic Reviews of Interventions* [83] in our evaluation of the methodology and hence the risk of bias of the included trials. We will evaluate the methodology in respect of the following:

- Random sequence generation
- Allocation concealment
- Blinding of participants and treatment providers
- Blinding of outcome assessment
- Incomplete outcome data
- Selective outcome reporting
- Other risk of bias
- Overall risk of bias

#### Random sequence generation

- *Low risk*: If sequence generation was achieved using computer random number generator or a random number table. Drawing lots, tossing a coin, shuffling cards, and throwing dice will also be considered adequate if performed by an independent adjudicator.
- *Unclear risk*: If the method of randomization was not specified, but the trial was still presented as being randomized
- *High risk*: If the allocation sequence was not randomized or only quasi-randomized. These trials will be excluded.

#### Allocation concealment

- *Low risk*: If the allocation of patients was performed by a central independent unit, on-site locked computer, identical-looking numbered sealed envelopes, or containers prepared by an independent investigator
- *Uncertain risk*: If the trial was classified as randomized but the allocation concealment process was not described
- *High risk*: If the allocation sequence was familiar to the investigators who assigned participants

#### Blinding of participants and treatment providers

- *Low risk*: If the participants and the treatment providers were blinded to intervention allocation and this was described
- *Uncertain risk*: If the procedure of blinding was insufficiently described
- *High risk*: If blinding of participants and the treatment providers was not performed

#### Blinding of outcome assessment

- *Low risk of bias*: If it was mentioned that outcome assessors were blinded and this was sufficiently described
- *Uncertain risk of bias*: If it was not mentioned if the outcome assessors in the trial were blinded or the extent of blinding was insufficiently described
- *High risk of bias*: If no blinding or incomplete blinding of outcome assessors was performed

#### Incomplete outcome data

- *Low risk of bias*: If missing data were unlikely to make treatment effects depart from plausible values. This could be either (1) there were no drop-outs or withdrawals for all outcomes or (2) the numbers and reasons for the withdrawals and drop-outs for all outcomes were clearly stated and could be described as being similar to both groups. Generally, the trial is judged as at a low risk of bias due to incomplete outcome data if drop-outs are less than 5%. However, the 5% cut-off is not definitive.
- *Uncertain risk of bias*: If there was insufficient information to assess whether missing data were likely to induce bias on the results.
- *High risk of bias*: If the results were likely to be biased due to missing data either because the pattern of drop-outs could be described as being different in the two intervention groups or the trial used improper methods in dealing with the missing data (e.g., last observation carried forward).

#### Selective outcome reporting

- *Low risk of bias*: If a protocol was published before or at the time the trial begun and the outcomes specified in the protocol were reported on
- *Uncertain risk of bias*: If no protocol was published
- *High risk of bias*: If the outcomes in the protocol were not reported on

#### Other risk of bias

- *Low risk of bias*: If the trial appears to be free of other components (for example, academic bias or for-profit bias) that could put it at risk of bias
- *Unclear risk of bias*: If the trial may or may not be free of other components that could put it at risk of bias
- *High risk of bias*: If there are other factors in the trial that could put it at risk of bias (for example, authors conducted trials on the same topic, for-profit bias)

#### Overall risk of bias

- *Low risk of bias*: The trial will be classified as overall “low risk of bias” only if all of the bias domains described in the above paragraphs are classified as low risk of bias.
- *High risk of bias*: The trial will be classified as “high risk of bias” if any of the bias risk domains described above are classified as “unclear” or high risk of bias.

We will assess the domains “blinding of outcome assessment,” “incomplete outcome data,” and “selective outcome reporting” for each outcome result. Thus, we can assess the bias risk for each outcome assessed in addition to each trial. Our primary conclusions will be based on the results of our primary outcome results with overall low risk of bias. Both our primary and secondary conclusions will be presented in the summary of findings tables.

#### Differences between protocol and the review

We will conduct the review according to this published protocol and report any deviations from it in the “Differences between the protocol and the review” section of the systematic review.

#### Measures of treatment effect

##### Dichotomous outcomes

We will calculate risk ratios (RRs) with 95% confidence interval (CI) for dichotomous outcomes, as well as the Trial Sequential Analysis-adjusted CIs (see below).

##### Continuous outcomes

We will calculate the mean differences (MDs) and consider calculating the standardized mean difference (SMD) with 95% CI for continuous outcomes. We will also calculate trial sequential analysis-adjusted CIs (see below).

#### Dealing with missing data

We will, as the first option, contact all trial authors to obtain any relevant missing data (i.e., for data extraction and for assessment of risk of bias, as specified above).

##### Dichotomous outcomes

We will not impute missing values for any outcomes in our primary analysis. In our sensitivity analyses (see paragraph below), we will impute data.

##### Continuous outcomes

We will primarily analyze scores assessed at single time points. If only changes from baseline scores are reported, we will analyze the results together with follow-up scores [83]. If standard deviations (SDs) are not reported, we will calculate the SDs using trial data, if possible. We will not use intention-to-treat data if the original report did not contain such data. We will not impute missing values for any outcomes in our primary analysis. In our sensitivity analysis (see paragraph below) for continuous outcomes, we will impute data.

#### Assessment of heterogeneity

We will primarily investigate forest plots to visually assess any sign of heterogeneity. We will secondly assess the presence of statistical heterogeneity by chi^2^ test (threshold *P* < 0.10) and measure the quantities of heterogeneity by the *I*^*2*^ statistic [87, 88]. We will investigate possible heterogeneity through subgroup analyses. We may ultimately decide that a meta-analysis should be avoided [83].

#### Assessment or reporting biases

We will use a funnel plot to assess reporting bias if ten or more trials are included. We will visually inspect funnel plots to assess the risk of bias. We are aware of the limitations of a funnel plot (i.e., a funnel plot assesses bias due to small sample size). From this information, we assess possible reporting bias. For dichotomous outcomes, we will test asymmetry with the Harbord test [89] if *τ*^2^ is less than 0.1 and with the Rücker test if *τ*^2^ is more than 0.1. For continuous outcomes, we will use the regression asymmetry test [90] and the adjusted rank correlation [91].

##### Unit of analysis issues

We will only include randomized clinical trials. For trials using crossover design, only data from the first period will be included [83, 92]. There will therefore not be any unit of analysis issues. We will not include cluster randomized trials.

#### Data synthesis

##### Meta-analysis

We will undertake the meta-analysis according to the recommendations stated in the *Cochrane Handbook for Systematic Reviews of Interventions* [83], Keus et al. [93], and the eight-step assessment suggested by Jakobsen et al. [94]. We will use the statistical software Stata version 16 [85] to analyze data. We will assess our intervention effects with both random-effects meta-analyses [95] and fixed-effects meta-analyses [96]. We will use the more conservative point estimate of the two [94]. The more conservative point estimate is the estimate closest to zero effect. If the two estimates are similar, we will use the estimate with the widest CI. We assess a total of six primary and secondary outcomes, and we will therefore consider a *P* value of 0.014 or less as the threshold for statistical significance [94]. We will investigate possible heterogeneity through subgroup analyses. Ultimately, we may decide that a meta-analysis should be avoided [83]. We will use the eight-step procedure to assess if the thresholds for significance are crossed [94]. Our primary conclusion will be based on results with low risk of bias [94]. Where multiple trial arms are reported in a single trial, we will include only the relevant arms. If two comparisons are combined in the same meta-analysis, we will halve the control group to avoid double-counting [83]. Trials with a factorial design will be included. In case of, e.g., a 2 × 2 factorial designed trial, the two groups receiving short-term interventions will be considered short-term control groups, while the two groups receiving long-term control interventions will be considered long-term control groups. If quantitative synthesis is not appropriate due to considerable heterogeneity or a small number of included trials, we will report the results in a narrative way.

##### Trial sequential analysis

Traditional meta-analysis runs the risk of random errors due to sparse data and repetitive testing of accumulating data when updating reviews. We wish to control the risks of type I errors and type II errors. We will therefore perform Trial Sequential Analysis on the outcomes, in order to calculate the required information size (that is, the number of participants needed in a meta-analysis to detect or reject a certain intervention effect) and the cumulative *Z*-curve’s breach of relevant trial sequential monitoring boundaries [84, 97–104]. A more detailed description of trial sequential analysis can be found in the trial sequential analysis manual [103] and at http://www.ctu.dk/tsa/.

For dichotomous outcomes, we will estimate the required information size based on the observed proportion of patients with an outcome in the control group (the cumulative proportion of patients with an event in the control groups relative to all patients in the control groups), a relative risk reduction of 20%, an alpha of 1.4% for all our outcomes, a beta of 20%, and the observed diversity as suggested by the trials in the meta-analysis. For continuous outcomes, we will in the trial sequential analysis use the observed SD, a mean difference of the observed SD/2, an alpha of 1.4% for all outcomes, a beta of 20%, and the observed diversity as suggested by the trials in the meta-analysis.

#### Subgroup analysis and integration of heterogeneity

##### Subgroup analysis

We will perform the following subgroup analyses when analyzing the primary outcomes (quality of life, serious adverse events, and symptom severity).

1. High risk of bias trials compared to low risk of bias trials
2. Types of psychiatric disorders
3. Types of psychotherapy comparisons
4. Trials above and below the mean difference in intervention lengths

We will use the formal test for subgroup interactions in Stata [85].

##### Sensitivity analysis

To assess the potential impact of the missing data for dichotomous outcomes, we will perform the two following sensitivity analyses on both the primary and secondary outcomes.

- “*Best*-*worst*-*case*” *scenario*: We will assume that all participants lost to follow-up in the short-term experimental group had no serious adverse event, had no suicides, had no suicide attempts, and had no self-harm and that all those participants lost to follow-up in the long-term control group did not survive, had a serious adverse event, had a suicide attempt, and had at least one episode of self-harm.

- “*Worst*-*best*-*case*” *scenario*: We will assume that all participants lost to follow-up in the short-term control group did not survive, had serious adverse event, had a suicide attempt, and had at least one episode of self-harm and that all those participants lost to follow-up in the long-term control group have survived, had no serious adverse event, had no suicide attempts, and had no self-harm.

We will present results of both scenarios in our review. When analyzing quality of life, symptom severity, and level of functioning, a “beneficial outcome” will be the group mean plus two standard deviations (SDs) (we will secondly use one SD in another sensitivity analysis) of the group mean and a “harmful outcome” will be the group mean minus two SDs (we will secondly use one SD in another sensitivity analysis) of the group mean [94]. To assess the potential impact of missing SDs for continuous outcomes, we will perform the following sensitivity analysis.

- Where SDs are missing and it is not possible to calculate them, we will impute SDs from trials with similar populations and low risk of bias. If we find no such trials, we will impute SDs from trials with a similar population. As the final option, we will impute SDs from all trials.

We will present results of this scenario in our review. Other post hoc sensitivity analyses might be warranted if unexpected clinical or statistical heterogeneity is identified during the analysis of the review results [94].

##### “Summary of findings” table

We will create a summary of findings table using each of the prespecified outcomes (quality of life, serious adverse events, symptom severity, suicide and suicide attempts, self-harm, and level of functioning) We will use the five GRADE considerations (bias risk of the trials, consistency of effect, imprecision, indirectness, and publication bias) to assess the quality of a body of evidence as it relates to the studies which contribute data to the meta-analyses for the prespecified outcomes [94, 105–107]. We will assess imprecision using Trial Sequential Analysis. Otherwise, we will use methods and recommendations described in the *Cochrane Handbook for Systematic Reviews of Interventions* [83] using GRADEpro software. We will justify all decisions to downgrade the quality of studies using footnotes, and we will make comments to aid the reader’s understanding of the review where necessary. Firstly, we will present our results in the Summary of Findings table based on the results from the trials with low risk of bias, and secondly, we will present the results based on all trials.

## Discussion

This protocol aims at comparing the effects of short-term psychotherapy with the effects of long-term psychotherapy for common adult psychiatric disorders to determine the best length of treatment. The outcomes will be quality of life, serious adverse events, symptom severity, suicide or suicide attempts, self-harm, and level of functioning.

This protocol has a number of strengths. The predefined methodology is based on the *Cochrane Handbook for Systematic Reviews of Interventions* [83], the eight-step assessment suggested by Jakobsen et al. [94], Trial Sequential Analysis [84], and GRADE assessment [105–107]. Hence, this protocol considers both risks of random errors and risks of systematic errors. Another strength of this protocol is that we pragmatically compare two overall treatment strategies with each other, i.e., the results of this review will potentially reflect the effects of the two strategies in clinical everyday practice.

Our protocol also has some limitations. The primary limitation is the potential for large heterogeneity as a result of including all psychiatric disorders and all types of psychotherapy. Therefore, we may ultimately decide that a meta-analysis is not warranted. Further, psychotherapy always consists of multiple treatment elements and it is likely that different interventions have different effects. Hence, if we show a difference between the compared strategies, it will be difficult to conclude what exactly caused the difference in effect. To minimize this limitation, a number of subgroups are planned, but results of subgroup analyses should always be interpreted with great caution. Another limitation is the large number of comparisons which increase the risk of type 1 error. We have adjusted our thresholds for significance according to the number of primary outcomes, but as mentioned, we have also included multiple subgroup analyses. This large risk of type 1 error will be considered when interpreting the review results. Further, we expect that no trials will have blinded treatment providers and patients. Even though blinding of patients should be relatively easy, blinding of treatment providers is theoretically possible but much more difficult to carry out. Finally, we rely on the trialists defining their compared interventions as short-term and long-term (or similar terminology). Hence, we will not include trials comparing a short-term and a long-term version of the same psychotherapy type, if the trialists did not explicitly define their interventions with such terminology. Using trialists’ definitions of short-term and long-term psychotherapy potentially introduces problems with heterogeneity. However, we believe that our choice of methodology from a pragmatic point of view is the best solution there is. First, trialists often report poorly and often do not themselves use thresholds and important data might be excluded from our review if we demand exact definitions of lengths. Further, we do not expect to include many trials in this systematic review. Hence, relying on trialists definitions of short-term versus long-term psychotherapy may increase the number of trials being eligible for inclusion. Finally, we believe this pragmatic methodology will lead to the inclusion of the most relevant trials.

## Additional files

Additional file 1: PRISMA-P 2015 Checklist. (DOCX 30 kb)

Additional file 2: Search strategies. (DOC 46 kb)

## Data Availability

Data sharing is not applicable to this protocol article. We will publish all data including code in the supplementary material of the systematic review.

## Abbreviations

ABFT: Attachment-based family therapy
ACT: Acceptance and commitment therapy
ADHD: Attention deficit hyperactivity disorder
CAT: Cognitive-analytic therapy
CBT: Cognitive behavioral therapy
CENTRAL: Cochrane Central Register of Controlled Trials
CFT: Compassion-focused therapy
CI: Confidence interval
CPCI-S: Conference Proceedings Citation Index—Science
CPCI-SSH: Conference Proceedings Citation Index—Social Science & Humanities
DSM-V: Diagnostic and statistical manual of mental disorders, 5th edition
EMA: European Medicines Agency
EMBASE: Excerpta Medica database
GRADE: The Grading of Recommendations Assessment, Development and Evaluation
ICD-10: International Classification of Diseases and Related Health Problems – 10th edition
ICH-GCP: International Conference on Harmonisation of technical requirements for registration of pharmaceuticals for human use – Good Clinical Practice
ICTRP: International Clinical Trials Registry Platform
MD: Mean differences
MEDLINE: Medical Literature Analysis and Retrieval System Online
PRISMA: Preferred reporting items for systematic review and meta-analysis
PRISMA-P: Preferred reporting items for systematic review and meta-analysis – protocols
PROSPERO: International Prospective Register of Systematic Reviews
PTSD: Post-traumatic stress disorder
RR: Risk ratio
SCI-EXPANDED: Science index citation expanded
SD: Standard deviation
SFT: Schema-Focused Therapy
SMD: Standardized mean difference
SSCI: Social Science Citation Index
TFP: Transference-focused psychotherapy
TRIP: Turning research into practice
WHO: World Health Organization
