# Supplementary material for: Short-term versus long-term psychotherapy for adult psychiatric disorders: a protocol for a systematic review with meta-analysis and trial sequential analysis
Source: Syst Rev. 2019 Jul 13;8:169. doi: 10.1186/s13643-019-1099-0 (PMC6626421; doi:10.1186/s13643-019-1099-0)
Supplement: Supplementary file 2 — Search strategies. (DOC 46 kb) [file 13643_2019_1099_MOESM2_ESM.doc]

**Search strategies for**

**Short-term versus long-term psychotherapy for adult psychiatric disorders: a protocol for a systematic review with meta-analysis and Trial Sequential Analysis**

**(Juul et al.)**

**Cochrane Central Register of Controlled Trials (CENTRAL; 2019, Issue 6) in the Cochrane Library (1 hits)**

#1 MeSH descriptor: [Attention Deficit Disorder with Hyperactivity] explode all trees

#2 (attention deficit hyperactivity disorder or adhd).ti,ab

#3 #1 or #2

#4 MeSH descriptor: [Psychotic Disorders] explode all trees

#5 MeSH descriptor: [Schizophrenia] explode all trees

#6 (psychotic or delusion* or hallucination* or ((disorgani*ed or abnormal) near (think* or motor*)) or schizophreni*).ti,ab

#7 #4 or #5 or #6

#8 MeSH descriptor: [Bipolar Disorder] explode all trees

#9 (bipolar or mood elevation or mania or hypomania or depress*).ti,ab

#10 #8 or #9

#11 MeSH descriptor: [Depressive Disorder] explode all trees

#12 (depressi* or mood or unipolar).ti,ab

#13 #11 or #12

#14 MeSH descriptor: [Anxiety Disorders] explode all trees

#15 (anxiet* or fear or avoidance behavior* or phobia* or panic* or agoraphobia).ti,ab

#16 #14 or #15

#17 MeSH descriptor: [Obsessive-Compulsive Disorder] explode all trees

#18 (obsessive compulsive disorder or OCD or urge* or obsessi* or (repetetive near (mental or behavior*))).ti,ab

#19 #17 or #18

#20 MeSH descriptor: [Stress Disorders, Post-Traumatic] explode all trees

#21 (post-trauma* or trauma*).ti,ab

#22 #20 or #21

#23 MeSH descriptor: [Feeding and Eating Disorders] explode all trees

#24 (eating behavior or anorexia* or bulimia* or binge-eating*).ti,ab

#25 #23 or #24

#26 MeSH descriptor: [Personality Disorders] explode all trees

#27 (schizotypal* or paranoid* or schizoid* or histrionic* or narcissistic* or antisocial* or borderline* or avoidant* or dependent or obsessive-compulsi*).ti,ab

#28 #26 or #27

#29 3 or 7 or 10 or 13 or 16 or 19 or 22 or 25 or 28

#30 MeSH descriptor: [Psychotherapy] explode all trees

#31 (((psycho* or cognitive or behavior* or humanistic or systemic) and therap*) or psychotherap* or self care or self-care).ti,ab

#32 #30 or #31

#33 (brief or extended or standard or intensiv* or ((short* or long*) and term)).ti,ab

#34 #32 and #33

#35 #29 and #34

**MEDLINE Ovid (1946 to June 2019) (5249 hits)**1. exp Attention Deficit Disorder with Hyperactivity/

2. (attention deficit hyperactivity disorder or adhd).ti,ab.

3. 1 or 2

4. exp Psychotic Disorders/

5. exp Schizophrenia/

6. (psychotic or delusion* or hallucination* or ((disorgani*ed or abnormal) adj (think* or motor*)) or schizophreni*).ti,ab.

7. 4 or 5 or 6

8. exp Bipolar Disorder/

9. (bipolar or mood elevation or mania or hypomania or depress*).ti,ab.

10. 8 or 9

11. exp Depressive Disorder/

12. (depressi* or mood or unipolar).ti,ab.

13. 11 or 12

14. exp Anxiety Disorders/

15. (anxiet* or fear or avoidance behavior* or phobia* or panic* or agoraphobia).ti,ab.

16. 14 or 15

17. exp Obsessive-Compulsive Disorder/

18. (obsessive compulsive disorder or OCD or urge* or obsessi* or (repetetive adj (mental or behavior*))).ti,ab.

19. 17 or 18

20. exp Stress Disorders, Post-Traumatic/

21. (post-trauma* or trauma*).ti,ab.

22. 20 or 21

23. exp "Feeding and Eating Disorders"/

24. (eating behavior or anorexia* or bulimia* or binge-eating*).ti,ab.

25. 23 or 24

26. exp Personality Disorders/

27. (schizotypal* or paranoid* or schizoid* or histrionic* or narcissistic* or antisocial* or borderline* or avoidant* or dependent or obsessive-compulsi*).ti,ab.

28. 26 or 27

29. 3 or 7 or 10 or 13 or 16 or 19 or 22 or 25 or 28

30. exp psychotherapy/

31. (((psycho* or cognitive or behavior* or humanistic or systemic) and therap*) or psychotherap* or self care or self-care).ti,ab.

32. 30 or 31

33. (brief or extended or standard or intensiv* or ((short* or long*) and term)).ti,ab.

34. 32 and 33

35. 29 and 34

36. limit 35 to ("all adult (19 plus years)" or "adolescent (13 to 18 years)" or "young adult (19 to 24 years)" or "adult (19 to 44 years)" or "young adult and adult (19-24 and 19-44)" or "middle age (45 to 64 years)" or "middle aged (45 plus years)" or "all aged (65 and over)" or "aged (80 and over)")

37. (random* or blind* or placebo* or meta-analys*).mp. [mp=title, abstract, original title, name of substance word, subject heading word, floating sub-heading word, keyword heading word, organism supplementary concept word, protocol supplementary concept word, rare disease supplementary concept word, unique identifier, synonyms]

38. 36 and 37

**Embase Ovid (1974 to June 2019) (5891 hits)**

1. exp attention deficit disorder/

2. (attention deficit hyperactivity disorder or adhd).ti,ab.

3. 1 or 2

4. exp psychosis/

5. exp schizophrenia/

6. (psychotic or delusion* or hallucination* or ((disorgani*ed or abnormal) adj (think* or motor*)) or schizophreni*).ti,ab.

7. 4 or 5 or 6

8. exp bipolar disorder/

9. (bipolar or mood elevation or mania or hypomania or depress*).ti,ab.

10. 8 or 9

11. exp depression/

12. (depressi* or mood or unipolar).ti,ab.

13. 11 or 12

14. exp anxiety disorder/

15. (anxiet* or fear or avoidance behavior* or phobia* or panic* or agoraphobia).ti,ab.

16. 14 or 15

17. exp obsessive compulsive disorder/

18. (obsessive compulsive disorder or OCD or urge* or obsessi* or (repetetive adj (mental or behavior*))).ti,ab.

19. 17 or 18

20. exp posttraumatic stress disorder/

21. (post-trauma* or trauma*).ti,ab.

22. 20 or 21

23. exp eating disorder/

24. (eating behavior or anorexia* or bulimia* or binge-eating*).ti,ab.

25. 23 or 24

26. exp personality disorder/

27. (schizotypal* or paranoid* or schizoid* or histrionic* or narcissistic* or antisocial* or borderline* or avoidant* or dependent or obsessive-compulsi*).ti,ab.

28. 26 or 27

29. 3 or 7 or 10 or 13 or 16 or 19 or 22 or 25 or 28

30. exp psychotherapy/

31. (((psycho* or cognitive or behavior* or humanistic or systemic) and therap*) or psychotherap* or self care or self-care).ti,ab.

32. 30 or 31

33. (brief or extended or standard or intensiv* or ((short* or long*) and term)).ti,ab.

34. 32 and 33

35. 29 and 34

36. limit 35 to (adult <18 to 64 years> or aged <65+ years>)

37. (random* or blind* or placebo* or meta-analys*).mp. [mp=title, abstract, heading word, drug trade name, original title, device manufacturer, drug manufacturer, device trade name, keyword, floating subheading word, candidate term word]

38. 36 and 37

**LILACS (Bireme; 1982 to June 2019) (1163 hits)**

(attention deficit hyperactivity disorder or adhd) or (psychotic or delusion$ or hallucination$ or ((disorgani$ed or abnormal) and (think$ or motor$)) or schizophreni$) or (bipolar or mood elevation or mania or hypomania or depress$) or (depressi$ or mood or unipolar) or (anxiet$ or fear or avoidance behavior$ or phobia$ or panic$ or agoraphobia) or (obsessive compulsive disorder or OCD or urge$ or obsessi$ or (repetetive and (mental or behavior$))) or (post-trauma$ or trauma$) or (eating behavior or anorexia$ or bulimia$ or binge-eating$) or (schizotypal$ or paranoid$ or schizoid$ or histrionic$ or narcissistic$ or antisocial$ or borderline$ or avoidant$ or dependent or obsessive-compulsi$) [Words] and (((psycho$ or cognitive or behavior$ or humanistic or systemic) and therap$) or psychotherap$ or self care or self-care) [Words] and (brief or extended or standard or intensiv$ or ((short$ or long$) and term)) [Words]

**Science Citation Index Expanded (SCI-EXPANDED) (1900 to June 2019); Social Sciences Citation Index (SSCI) (1956 to June 2019); Conference Proceedings Citation Index- Science (CPCI-S) (1990 to June 2019); and Conference Proceedings Citation Index- Social Science & Humanities (CPCI-SSH) (1990 to June 2019) (Web of Science) (5401 hits)**

#7 #6 AND #5

#6 TS=(random* or blind* or placebo* or meta-analys*)

#5 #4 AND #1

#4 #3 AND #2

#3 TS=(brief or extended or standard or intensiv* or ((short* or long*) and term))

#2 TS=(((psycho* or cognitive or behavior* or humanistic or systemic) and therap*) or psychotherap* or self care or self-care)

#1 TI=((attention deficit hyperactivity disorder or adhd) or (psychotic or delusion* or hallucination* or ((disorgani*ed or abnormal) and (think* or motor*)) or schizophreni*) or (bipolar or mood elevation or mania or hypomania or depress*) or (depressi* or mood or unipolar) or (anxiet* or fear or avoidance behavior* or phobia* or panic* or agoraphobia) or (obsessive compulsive disorder or OCD or urge* or obsessi* or (repetetive near (mental or behavior*))) or (post-trauma* or trauma*) or (eating behavior or anorexia* or bulimia* or binge-eating*) or (schizotypal* or paranoid* or schizoid* or histrionic* or narcissistic* or antisocial* or borderline* or avoidant* or dependent or obsessive-compulsi*))
